# Valid comparisons and decisions based on clinical registers and population based cohort studies: assessing the accuracy, completeness and epidemiological relevance of a breast cancer query database

**DOI:** 10.1186/1756-0500-5-700

**Published:** 2012-12-27

**Authors:** Christian Olaf Jacke, Mathias Kalder, Uwe Wagner, Ute-Susann Albert

**Affiliations:** 1 Mental Health Services Research Group, Central Institute of Mental Health, Medical Faculty Mannheim / Heidelberg University, Mannheim, Germany; 2 Department of Gynecology, Gynecological Endocrinology and Oncology, Breast Center Regio, University of Marburg, Marburg, Germany

**Keywords:** Data quality, Data quality indicators (DQI), Data correctness, Data accuracy, Data completeness

## Abstract

**Background:**

Data accuracy and completeness are crucial for ensuring both the correctness and epidemiological relevance of a given data set. In this study we evaluated a clinical register in the administrative district of Marburg-Biedenkopf, Germany, for these criteria.

**Methods:**

The register contained data gathered from a comprehensive integrated breast-cancer network from three hospitals that treated all included incident cases of malignant breast cancer in two distinct time periods from 1996–97 (N=389) and 2003–04 (N=488). To assess the accuracy of this data, we compared distributions of risk, prognostic, and predictive factors with distributions from established secondary databases to detect any deviations from these “true” population parameters. To evaluate data completeness, we calculated epidemiological standard measures as well as incidence-mortality-ratios (IMRs).

**Results:**

In total, 12% (13 of 109) of the variables exhibited inaccuracies: 9% (5 out of 56) in 1996–97 and 15% (8 out of 53) in 2003–04. In contrast to raw, unstandardized incidence rates, (in-) directly age-standardized incidence rates showed no systematic deviations. Our final completeness estimates were IMR=36% (1996–97) and IMR=43% (2003–04).

**Conclusion:**

Overall, the register contained accurate, complete, and correct data. Regional differences accounted for detected inaccuracies. Demographic shifts occurred. Age-standardized measures indicate an acceptable degree of completeness. The IMR method of measuring completeness was inappropriate for incidence-based data registers. For the rising number of population-based health-care networks, further methodological advancements are necessary. Correct and epidemiologically relevant data are crucial for clinical and health-policy decision-making.

## Background

Today^′^s information technologies make it possible to collect and process comprehensive population-based public-health data. Cohort studies and data register collect, check, and analyze medical data in regular time intervals. This procedure is intended to provide high-quality data on which crucial health-policy decisions can be based. The goal of this so-called “public-disclosure strategy” is to increase health care system transparency of and for all stakeholders related to public health [1,2]. Increased transparency is important for various stakeholders (patients, physicians, hospital management, health insurers, clinical-trial coordinators, policymakers, etc.) in different settings (primary care, in-home care, biodatabases, etc.) and on various levels (micro, meso and macro) with regard to aspects of health care quality (structures, processes, outcomes) [3,4]. In all of the cases above, it is the quality of the data, as well as its verification and assessment that determine the quality of the subsequent information and decisions. Thus, decisions can never be better than the information retrieved from the data they are based on.

### The common conception of data quality

But what does “data quality” really mean? The scientific community has established standards such as objectivity, reliability, and validity of measurement instruments to ensure reproducible and comparable research results [5-7]. There have been, for example, various exploratory data-analysis methodologies in common use, each offering an independent method for achieving these standards [8,9]. In an effort to standardize these various methods, cancer registers have published extensive manuals of these numerous methods [10] to improve the validity of the comparable epidemiological indices used for health-monitoring reports. However, these manuals do not make theoretical distinctions between levels of data quality and they do not offer an integrative framework for explorative methods. To overcome these shortcomings the “guidelines for the adaptive management of data quality for cohort studies and registers” (GAMOQ)[6,7,11] were developed.

### The level-based conception of data quality

These GAMOQ were developed in 2006 by the technology and methodology platform for networked medical research (TMF) to facilitate the independent assessment of data quality as well as its subsequent improvement. An extensive literature review and expert interviews were part of the development process [7]. The GAMOQ differentiated the term “data quality” into three distinct levels – data plausibility, data organization, and data correctness – corresponding to the already-existing assessment approaches for quality of medical care (structure, process, and outcome quality) [4,12]. Each of these data levels encompasses specific data-quality indicators (DQIs). In total there are 24 DQIs which assess different aspects of a data set or register. A general proof of feasibility, usefulness, and practicability of this methodological framework has already been reported elsewhere [13,14].

### Data accuracy and completeness as objectives

Here we aim to present the most important results concerning data-level correctness of a population-based clinical breast-cancer registry from the longitudinal point of view. Of particular interest are the DQIs accuracy and completeness, both of which allow classifying a data set′s epidemiological relevance. In the following, accuracy refers to the degree to which the primary data differ from the population′s “true” parameters determined by using established secondary data sources [7]. Completeness refers to the degree to which the primary data have captured all relevant patients in accordance with the inclusion criteria [7]. The leading hypothesis assumes that the current population-based breast-cancer register is accurate and complete and that it contains epidemiologically relevant data.

## Methods

### Primary database

The primary data to be assessed was collected in three hospitals in the administrative district of Marburg-Biedenkopf, Germany. They included all females that were treated in these hospitals for the first time for malignant breast cancer (ICD-10: C.50). Recruiting was carried out within the context of two prospective cohort studies that took place in the periods from 1996–97 and 2003–04. The population surveys were conducted independently of one another, and thus the data was not processed according to a uniform standard. It was saved in different file formats (Excel, SPSS). In total, 1,389 (1996–97) and 150 variables (2003–04) related to demographic, socioeconomic, and medical issues were collected. The variable names and value codes of the two data sets′ variables were synchronized. The data sets were transferred to a clinical register called the Breast Cancer Query Database (BCQDB). The accuracy and completeness of the BCQDB was assessed. The study was approved and conducted according to the guidelines of the local ethics committee of the Philipps University Marburg (Germany).

### Secondary databases

Additional secondary data sources with high data-quality standards were used to estimate the epidemiological accuracy and completeness of the BCQDB. The “National Field Study for the Assessment of the Quality of Breast Cancer Care” [15] provided distributions and parameter estimates for risk, prognostic, and predictive factors for breast cancer, as did the common cancer registers of the German federal states of Berlin, Brandenburg, Mecklenburg-Vorpommern, Saxony-Anhalt, Saxony, and Thuringia [16,17], as well as the Munich Tumor Register [18]. For comparison purposes, we also integrated raw and age standardized incidence rates from the epidemiological cancer register of Saarland, Germany, and the German Center for Cancer Registry Data located at the Robert Koch Institute (RKI). To estimate mortality-incidence ratios, we integrated breast-cancer-specific mortality rates kept by the regional statistic authorities of the German federal state of Hesse.

### Measures of data accuracy

The central idea of the GAMOQ is to assess data quality, if necessary by source data verification techniques, by comparing the dataset for a cohort study or a data register (in this case, the BCQDB) with the original data files (in this case, the Excel and SPSS files). However, the latter can also be substituted by reliable external data if it is the data′s accuracy and completeness that are being assessed. Therefore, we measured the accuracy of the BCQDB register by comparing all available risk, prognostic, and predictive factors to distributions available from external databases included in this study (see above). These factors included patients′ age at breast-cancer diagnosis, tumor location, tumor size (pT), node status (pN), stage of the disease according to the Union for International Cancer Control (UICC), grading, and hormone-receptor status [19-21].

### Measures of data completeness

To critically appraise the BCQDB register′s completeness, established epidemiological measures were used [5,22-24]. We calculated raw age-specific incidence rates and cumulative incidence curves in order to identify any possible systematic deviations from external databases. We also calculated direct age-standardized incidence rates for the standard populations of both Europe and the world in order to account for any possible age-related or demographic differences. In addition, we estimated indirect age-standardized incidence rates, taking the population of the German federal state of Saarland as the standard population. This allowed the direct comparison between Saarland and the administrative district of Marburg-Biedenkopf without using neutral standard populations (e.g., Europe, the world). This approach allowed us to account for the demographic particularities of the Marburg-Biedenkopf administrative district, which is the catchment area of the BCQDB register. Finally, incidence-mortality ratios (IMR) were calculated for each year to obtain further insights into the BCQDB′s completeness [16,25,26].

### Statistics

Rates and ratios were calculated for the BCQDB and then compared to the corresponding figures for the various external databases. This statistical comparison was performed using an exact binomial test with the specified “true” parameters of the selected secondary databases. The probability of error of alpha was 0.05 [8,27]. The corresponding 95% confidence intervals were derived using Pearson-Clopper values which were based on F-distributions and which are appropriate for small samples [7] characteristically surveyed in rural areas (small-area analysis) [28]. Incidence-based parameters and distributions of risk, prognostic, and predictive factors were compared using overlapping 95% confidence intervals based on Gaussian normal distributions [23,24].

## Results

The survey recruited a total of *N*=877 patients (1996–97: *N*=389, 2003–04: *N*=488) who were primarily treated in the German administrative district of Marburg-Biedenkopf. Of these incident patients *N*=577 (1996–97: *N*=266, 2003–04: *N*=311) or 67% were registered as residents of that district and belonged to the epidemiologically relevant sample. The comparison of epidemiological parameters and distributions with external databases was only valid for residents. The BCQDB′s accuracy for the 1996–97 cohort is given in Table 1.

**Table 1:** Accuracy of primary data (BCQDB) in comparison to external databases, period 1996-97

	**Secondary data**	**Primary data**	**95% Confidence interval**		**Test-statistic**
**Variable-level**	**GER (θ**_**1**_**)**	**BCQDB (θ**_**2**_**)**	**Lower bound (θ**_**2**_**)**	**Upper bound (θ**_**2**_**)**	***p*-value**
00-49 years	21.4	24.4	19.4	30.1	ns
*50-69 years*	*50.5*	*43.6*	*37.6*	*49.8*	0.024
≥ 70 years	28.1	32.0	26.4	37.9	ns
pTis (non-invasive)	4.3	4.5	2.4	7.7	ns
pT1 (≤ 2cm)	47.4	53.0	46.8	59.1	ns
pT2 (≤ 5cm)	35.8	30.8	25.3	36.8	ns
pT3 (> 5cm)	4.7	2.6	1.1	5.3	ns
pT4 (involving other areals)	7.8	9.0	5.9	13.1	ns
pN+	37.0	32.7	27.1	38.7	ns
Involved lymph nodes 0	60.9	63.4	57.0	69.6	ns
Involved lymph nodes 1-3	20.1	19.3	14.5	24.9	ns
Involved lymph nodes 4-9	11.8	10.5	6.9	15.1	ns
Involved lymph nodes >10	7.2	6.7	3.9	10.7	ns
Stage 0	4.2	4.5	2.4	7.7	ns
Stage 1	36.0	38.7	32.8	44.9	ns
Stage 2a	28.5	27.8	22.5	33.6	ns
Stage 2b	16.4	12.0	8.4	16.6	ns
Stage 3a	4.2	2.3	0.8	4.8	ns
Stage 3b	6.3	8.6	5.6	12.7	ns
Stage 4	4.5	6.0	3.5	9.6	ns
*Grading 1 (G1)*	*11.3*	*7.9*	*4.9*	*11.9*	ns
*Grading 2 (G2)*	*55.8*	*43.3*	*37.1*	*49.6*	<0.001
*Grading 3 (G3)*	*c32.9*	*48.8*	*42.5*	*55.1*	<0.001
ERPR+	82.0	83.5	78.4	87.7	ns
Variable-level	GKR (θ_1_)	BCQDB (θ_2_)	Lower bound (θ_2_)	Upper bound (θ_2_)	p-value
00-49 years	20.4	24.4	19.4	30.1	ns
*50-69 years*	*49.0*	*43.6*	*37.6*	*49.8*	ns
≥ 70 years	30.6	32.0	26.4	37.9	ns
Left side	50.6	56.0	49.8	62.1	ns
Right side	47.7	44.0	37.9	50.2	ns
Breast quadrant I	48.6	46.6	40.5	52.8	ns
Breast quadrant II	12.3	11.3	7.7	15.7	ns
Breast quadrant III	9.1	11.7	8.1	16.1	ns
Breast quadrant IV	5.6	8.6	5.6	12.7	ns
Multifocal	16.6	17.3	12.9	22.4	ns
*Mamille*	*7.8*	*4.5*	*2.4*	*7.7*	0.045
Non-invasive tumor	5.8	4.5	2.4	7.7	ns
Stage 0	6.1	4.5	2.4	7.7	ns
Stage 1	38.4	38.7	32.8	44.9	ns
Stage 2	41.9	39.8	33.9	46.0	ns
Stage 3	8.0	10.9	7.4	15.3	ns
Stage 4	5.6	6.0	3.5	9.6	ns
Stage 0 | 0–49 years	7.0	7.7	2.5	17.0	ns
Stage 1 | 0–49 years	38.0	36.9	25.3	49.8	ns
Stage 2 | 0–49 years	43.7	46.2	33.7	59.0	ns
Stage 3 | 0–49 years	6.5	6.2	1.7	15.0	ns
Stage 4 | 0–49 years	4.8	3.1	0.4	10.7	ns
Stage 0 | 50–69 years	6.9	4.3	1.4	9.8	ns
Stage 1 | 50–69 years	39.4	41.4	32.3	50.9	ns
Stage 2 | 50–69 years	41.0	42.2	33.1	51.8	ns
Stage 3 | 50–69 years	7.7	6.9	3.0	13.1	ns
Stage 4 | 50–69 years	5.0	5.2	1.9	10.9	ns
Stage 0 | ≥70 years	4.1	2.4	0.3	8.2	ns
Stage 1 | ≥70 years	36.9	36.5	26.3	47.6	ns
Stage 2 | ≥70 years	42.2	31.8	22.1	42.8	ns
*Stage 3 | ≥70 years*	*9.7*	*20.0*	*12.1*	*30.1*	<0.001
Stage 4 | ≥70 years	7.1	9.4	4.2	17.7	ns

(θ) Population parameter in percent, (GER) Mean of a five German regions for 1996–97 (Engel et al., 2002).

(BCQDB) Breast Cancer Query Database, Marburg, Germany; (GKR) Common Cancer Registers of the states.

Berlin, Brandenburg, Mecklenburg-Vorpommern, Saxony-Anhalt, Saxony and Thuringa (Stabenow & Eisinger, 2001).

(pN+) Pathological assessment of metastases in lymph nodes, (ERPR) Estrogen-Progesterone-Receptors.

(Multifocal) All quadrants involved, (Stage | age) Percent of patients with stage number conditional on age groups.

Comparison with the external data sources revealed some isolated differences. These differences were randomly distributed over the included parameters and did not show any systematic patterns. Additional differences were also detected for the 2003–04 cohort (see Table 2).

**Table 2:** Accuracy of primary data (BCQDB) in comparison to external databases, period 2003-04

	**Secondard data**	**Primary data**	**95% Confidence interval**		**Test-statistic**
**Variable-level**	**TRM (θ**_**1**_**)**	**BCQDB ( θ**_**2**_**)**	**Lower bound (θ**_**2**_**)**	**Upper bound (θ**_**2**_**)**	***p*-value**
00-49 years	18.9	22.4	17.9	27.5	ns
*50-69 years*	*53.4*	*47.4*	*41.8*	*53.1*	0.035
≥ 70 years	27.6	30.1	25.1	35.6	ns
Involved lymph nodes 0	*61.1*	*68.4*	*62.7*	*73.8*	0.011
Involved lymph nodes 1-3	23.0	20.0	15.5	25.1	ns
Involved lymph nodes 4-9	9.9	7.4	4.6	11.0	ns
Involved lymph nodes >10	6.0	4.2	2.2	7.2	ns
pTis (non-invasive)	7.0	6.7	4.2	10.1	ns
pT1 (≤ 2cm)	53.0	53.8	48.1	59.5	ns
pT2 (≤ 5cm)	31.4	31.1	26.0	36.5	ns
pT3 (> 5cm)	3.9	2.9	1.3	5.4	ns
pT4 (involving other areals)	4.7	5.4	3.2	8.6	ns
pTis | 0–49 years	7.0	7.1	2.4	15.9	ns
pT1 | 0–49 years	57.5	64.3	51.9	75.4	ns
pT2 | 0–49 years	29.9	27.1	17.2	39.1	ns
pT3 | 0–49 years	3.4	1.4	0.0	7.7	ns
pT4 | 0–49 years*	1.5	-	-	-	-
pTis | 50–69 years	8.2	8.8	4.8	14.6	ns
pT1 | 50–69 years	57.2	56.1	47.7	64.2	ns
pT2 | 50–69 years	28.5	28.4	21.3	36.4	ns
pT3 | 50–69 years	3.3	3.4	1.1	7.7	ns
pT4 | 50–69 years	2.8	3.4	1.1	7.7	ns
pTis | ≥70 years	4.2	3.2	0.7	9.0	ns
pT1 | ≥70 years	41.9	42.6	32.4	53.2	ns
pT2 | ≥70 years	38.1	38.3	28.5	48.9	ns
pT3 | ≥70 years	5.4	4.3	1.2	10.5	ns
pT4 | ≥70 years	10.5	11.7	6.0	20.0	ns
Grading 1 (G1)	12.5	13.5	9.8	18.0	ns
Grading 2 (G2)	*55.2*	*72.0*	*66.4*	*77.1*	<0.001
Grading 3 (G3)	*32.3*	*14.5*	*10.7*	*19.1*	<0.001
Variable-level	GKR (θ_1_)	BCQDB ( θ_2_)	Lower bound (θ_2_)	Upper bound (θ_2_)	p-value
00-49 years	20.6	22.4	17.9	27.5	ns
50-69 years	49.8	47.4	41.8	53.1	ns
≥ 70 years	29.6	30.1	25.1	35.6	ns
pTis (non-invasive)	7.8	6.7	4.2	10.1	ns
*pT1 (≤ 2cm)*	*44.4*	*53.8*	*48.1*	*59.5*	<0.001
*pT2 (≤ 5cm)*	*36.7*	*31.1*	*26.0*	*36.5*	0.04
pT3 (> 5cm)	4.9	2.9	1.3	5.4	ns
pT4 (involving other areals)	6.3	5.4	3.2	8.6	ns
pTis | 0–49 years	8.4	7.1	2.4	15.9	ns
pT1 | 0–49 years	*46.8*	*64.3*	*51.9*	*75.4*	0.003
pT2 | 0–49 years	36.4	27.1	17.2	39.1	ns
pT3 | 0–49 years	5.1	1.4	0.0	7.7	ns
pT4 | 0–49 years	3.2	-	-	-	-
pTis | 50–69 years	9.0	8.8	4.8	14.6	ns
pT1 | 50–69 years	*47.6*	*56.1*	*47.7*	*64.2*	0.039
pT2 | 50–69 years	33.8	28.4	21.3	36.4	ns
pT3 | 50–69 years	4.5	3.4	1.1	7.7	ns
pT4 | 50–69 years	2.8	3.4	1.1	7.7	ns
pTis | ≥70 years	5.3	3.2	0.7	9.0	ns
pT1 | ≥70 years	37.3	42.6	32.4	53.2	ns
pT2 | ≥70 years	41.6	38.3	28.5	48.9	ns
pT3 | ≥70 years	5.5	4.3	1.2	10.5	ns
pT4 | ≥70 years	10.3	11.7	6.0	20.0	ns

(θ) Population parameter in percent, (TRM) Munich Tumor Register 11/2008 (Tumor Center Munich 2008), (BCQDB) Breast Cancer Query Register, Marburg, Germany, (GKR) Common Cancer Registers of the states Berlin, Brandenburg, Mecklenburg-Vorpommern, Saxony-Anhalt, Saxony and Thuringa (GKR, 2008). (Tumorsize | age) Percent of patients with tumorsize conditional on age groups, (−) No data.

**Figure 1:**
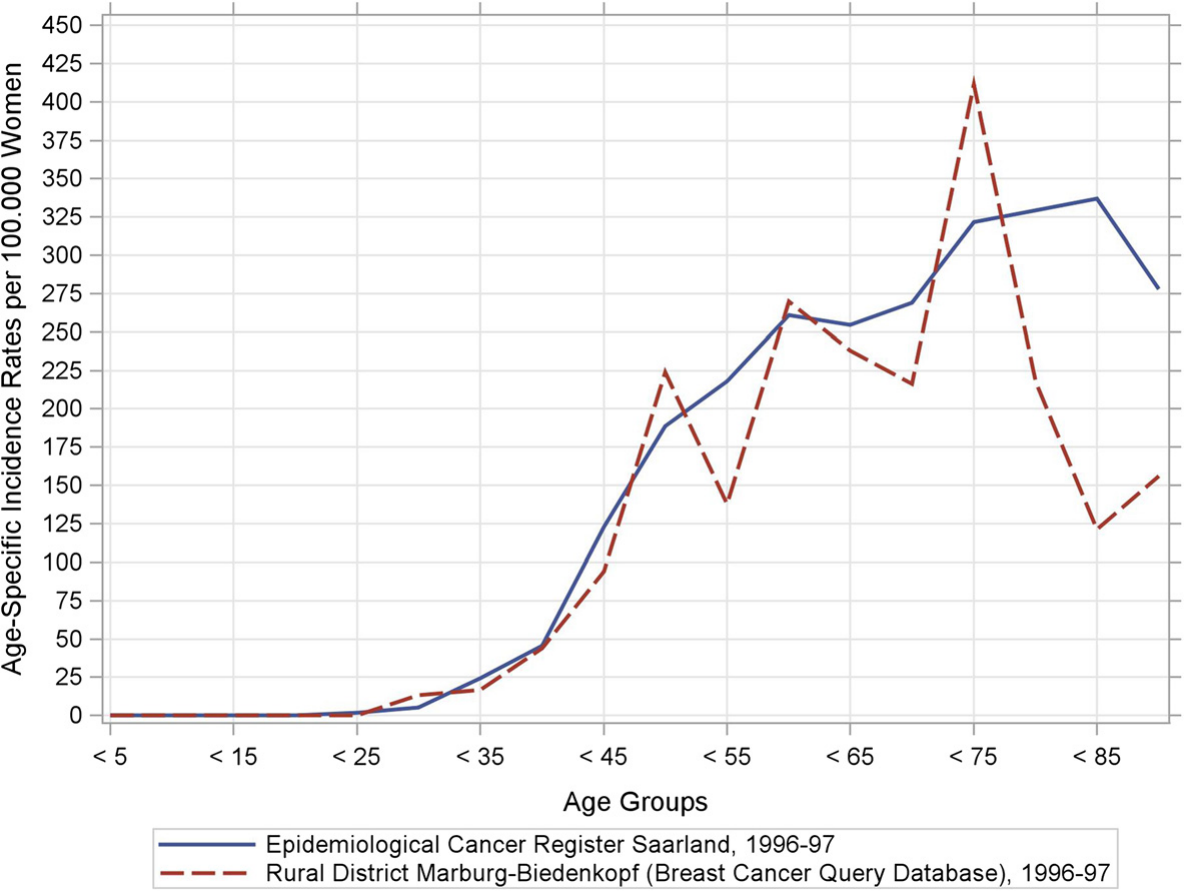
**Raw age-specific incidence rates and cumulative incidence rates from the breast cancer query database and the Saarland Epidemiological Cancer Register for 1996–97 and 2003–04.** Sub-figure (**a**): Raw incidence rates, 1996–97.

**Figure 2:**
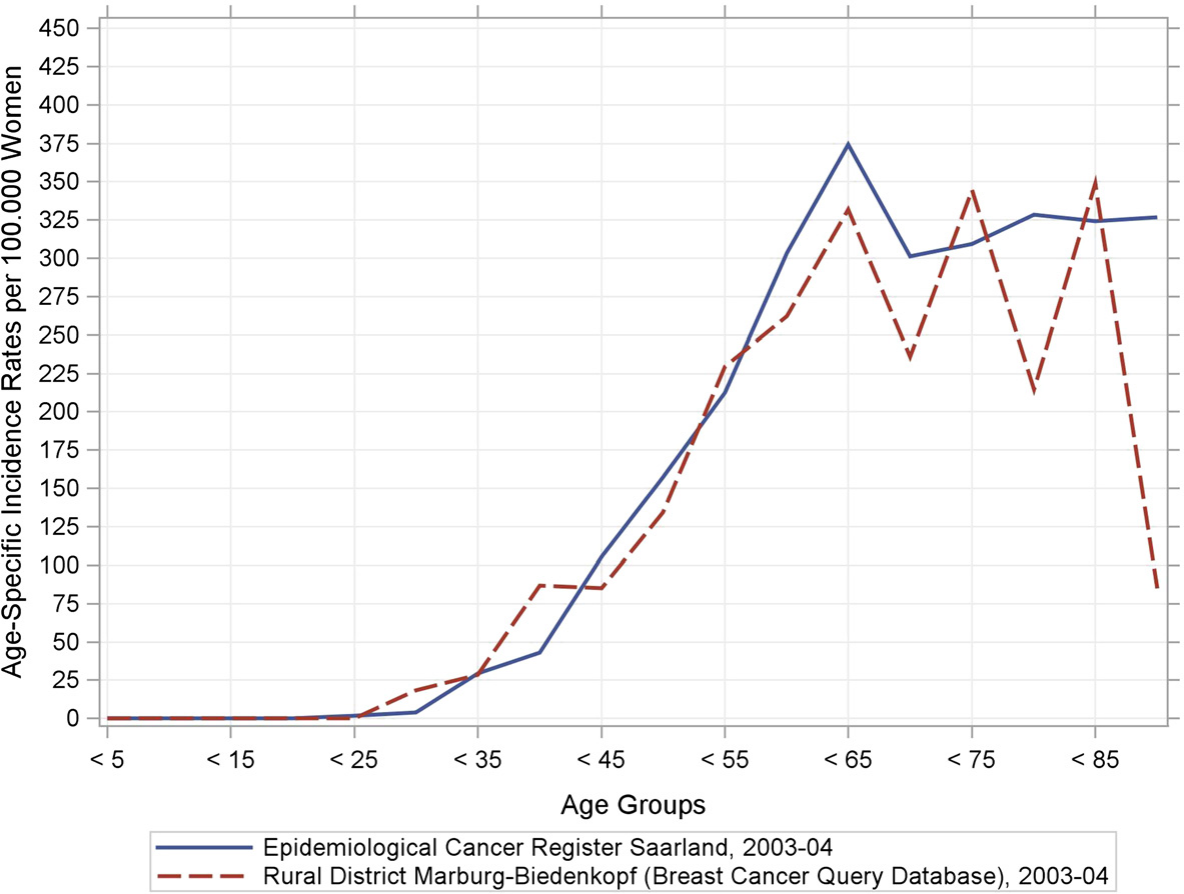
**Raw age-specific incidence rates and cumulative incidence rates from the breast cancer query database and the Saarland Epidemiological Cancer Register for 1996–97 and 2003–04.** Sub-figure (**b**): Raw incidence rates, 2003–04.

**Figure 3:**
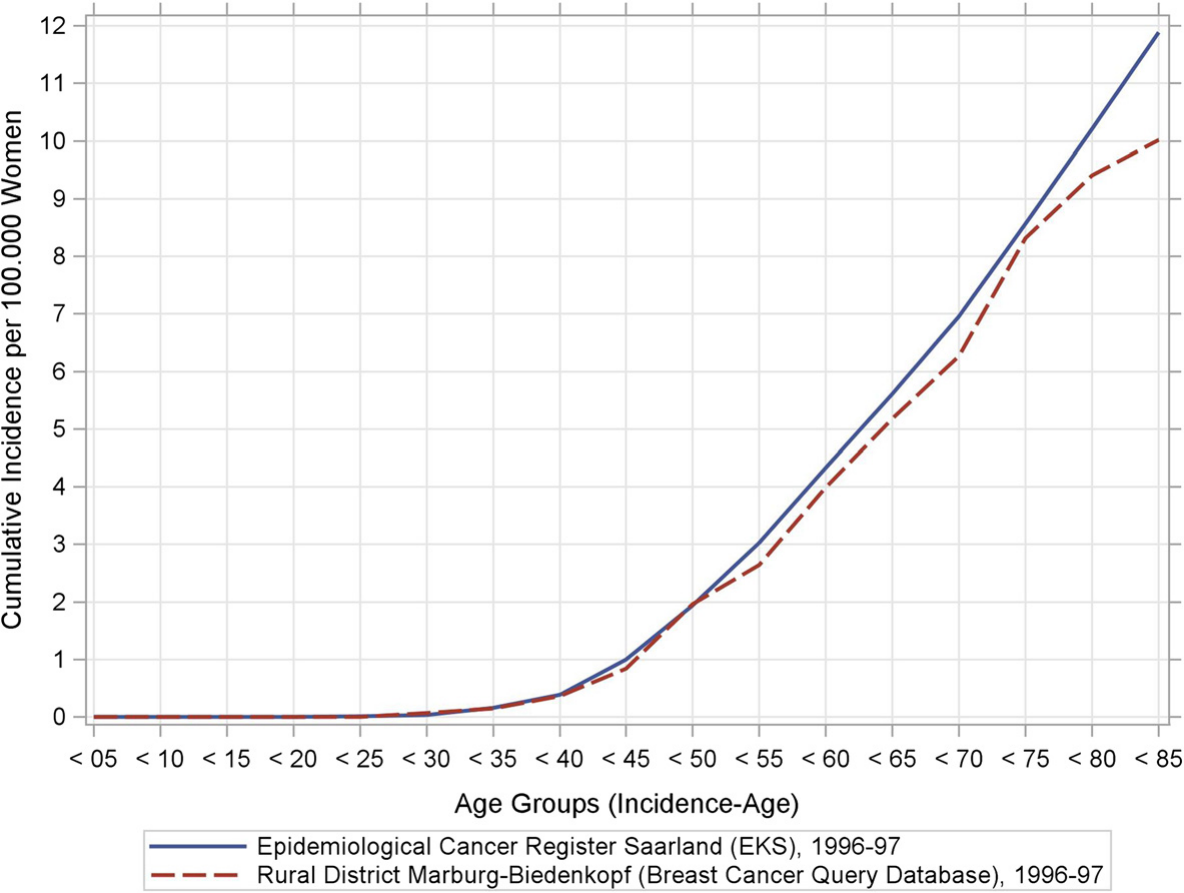
**Raw age-specific incidence rates and cumulative incidence rates from the breast cancer query database and the Saarland Epidemiological Cancer Register for 1996–97 and 2003–04.** Sub-figure (**c**): Cumulative incidence rates, 1996–97.

**Figure 4:**
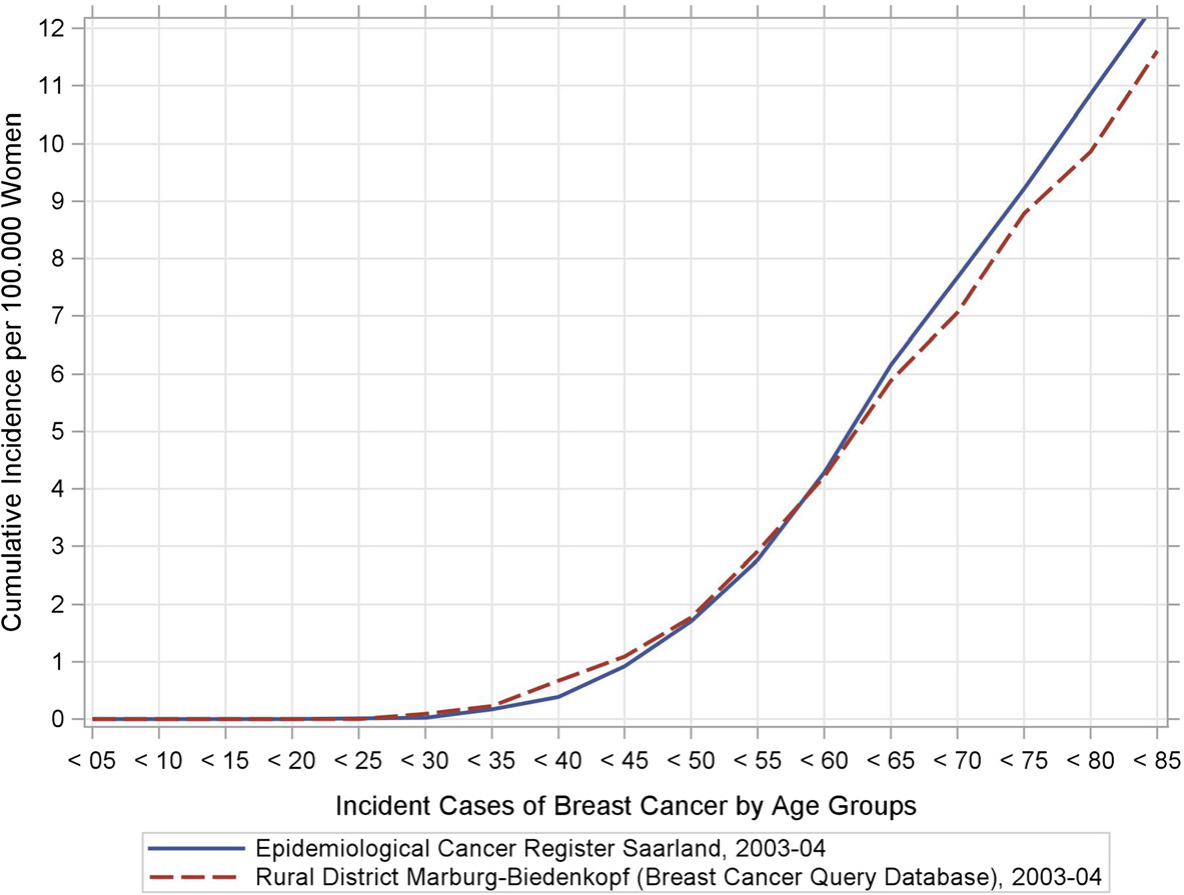
**Raw age-specific incidence rates and cumulative incidence rates from the breast cancer query database and the Saarland Epidemiological Cancer Register for 1996–97 and 2003–04.** Sub-figure (**d**): Cumulative incidence rates, 2003–04.

Table 2 indicates that the BCQDB register included more women under the age of 49 years as well as fewer patients in the 50–69 year range than the external data sources. Our stratified analyses of women′s age at breast-cancer diagnosis depending on the stage of the disease suggest that older women were under-represented in the BCQDB data. We checked this assumption using the raw, age-specific, and cumulative incidence curves. Figures 1, 2, 3, 4 show these results.

In the period from 1996–97, the age-specific incidence rates (Figure 1) already began to show deviations at age 50 years. These tended to scatter arbitrarily around the benchmark parameters. In the period from 2003–04, similar deviations occurred from the age of 65 years (Figure 2). Both BCQDB cohorts exhibited lower incidence rates than in the epidemiological cancer register of Saarland that begins approximately from the age of 75 years. The cumulative incidence rates (CIR), which showed greater deviations for the 1996–97 cohort than for the 2003–04 cohort (Figures 3, 4) conveyed a similar impression. The “kinks” at age 55 years in the 1996–97 cohort and at age 65 years in the 2003–04 cohort were especially revealing. The cumulative incidence rates were CIR (BCQBD, 1996–97) = 10.0 and CIR (BCQBD, 2003–04) = 11.6 in comparison to CIR (SAAR, 1996–97) = 11.9 and CIR (SAAR, 2003–04)= 12.5. However these differences were not apparent in the directly age-standardized incidence rates (SIR*, SIR**) or the indirectly standardized incidence ratios (SIR***). Table 3 shows these results.

**Table 3:** Age-standardized incidence rates for Germany, Saarland and Marburg-Biedenkopf

	**Germany**^**a**^	**Saarland**^**b**^			**Marburg-Biedenkopf**^**c**^		
			**95% Confidence level**			**95% Confidence level**	
**Year**	SIR*	SIR*	**Lower bound**	**Upper bound**	SIR*	**Lower bound**	**Upper bound**
1996	98.2	103.1	95.3	110.9	93.4	76.6	110.2
1997	99.2	104.9	96.9	112.8	92.9	76.7	109.3
2003	102.3	114.3	106.2	122.5	101.3	84.6	118.0
2004	102.3	105.9	98.1	113.7	101.7	84.9	118.4
	SIR**	SIR**	Lower bound	Upper bound	SIR**	Lower bound	Upper bound
1996	72.4	74.9	68.9	80.6	69.1	56.4	81.9
1997	73.1	76.9	70.9	82.9	70.4	57.7	83.2
2003	75.4	82.6	76.6	88.6	75.3	62.6	87.9
2004	75.4	77.5	71.6	83.4	74.8	62.2	87.4
					SIR***	Lower bound	Upper bound
1996					0.873	0.733	1,039
1997					0.884	0.744	1,049
2003					0.866	0.738	1,017
2004					0.938	0.799	1,099

(a) Point estimates provided by German Center for Cancer Register Data 12/2010, (b) Point estimates and confidence. Intervals by Epidemiological Cancer Register of Saarland, (c ) Point estimates and confidence intervals by Breast Cancer. Query Database, (SIR) Direct age-standardized incidence ratio using (*) the european or (**) world standard population. (SIR***) Indirect age-standardized incidence ratio using Saarland demographics as standard population.

As expected, we found no systematic differences in data completeness when using incidence rates that were age-standardized. In contrast, our estimations using incidence-mortality ratios (IMR) estimated completeness to be low: IMR (1996–97) = 36.8 and IMR (2003–04) = 43.8. Detailed results on these incidence-mortality ratios are given in Table 4.

**Table 4:** Mamma carcinoma incidence-mortality ratios for estimating completeness

	**Saarland**^**a**^			**Marburg-Biedenkopf**				
	**Incidence**	**Mortality**	**I:M Factor**	**Mortality**^**b**^	**Expected**^**c**^	**Observed**^**d**^	**Cumulative expected**	**Cumulative observed**
**Ages**	**1996-97**	**1996-97**	**1996-97**	**1996-97**	**1996-97**	**1996-97**	**1996-97**	**1996-97**
00 ≤ 25	0.5	0	-	0	-	0	-	0.0
25 ≤ 30	2.0	0	-	0	-	0	-	0.0
30 ≤ 35	11.0	0.5	22.0	2	44.0	2	44.0	2.0
35 ≤ 40	20.0	4.5	4.4	4	17.8	5	61.8	6.5
40 ≤ 45	49.0	10.5	4.7	4	18.7	8	80.4	14.5
45 ≤ 50	67.5	22.0	3.1	6	18.4	17	98.9	31.0
50 ≤ 55	62.0	14.5	4.3	10	42.8	8	141.6	39.0
55 ≤ 60	106.5	33.0	3.2	16	51.6	22	193.2	60.5
60 ≤ 65	89.0	30.5	2.9	6	17.5	16	210.8	76.0
65 ≤ 70	86.5	37.5	2.3	18	41.5	13	252.3	89.0
70 ≤ 75	105.0	30.5	3.4	12	41.3	25	293.6	113.5
75 ≤ 80	70.0	40.0	1.8	17	29.8	10	323.3	123.0
80 ≤ 85	54.0	29.5	1.8	14	25.6	4	349.0	127.0
85 and older	39.5	34.0	1.2	11	12.8	5	*361.7*	*131.5*
Period	2003-04	2003-04	2003-04	2003-04	2003-04	2003-04	2003-04	2003-04
0 ≤ 25	0.5	0	-	0	-	0	-	0.0
25 ≤ 30	1.0	0	-	1	-	0	-	0.0
30 ≤ 35	9.5	0.5	19.0	0	0.0	3	0	2.5
35 ≤ 40	18.5	6.5	2.8	3	8.5	10	8.5	12.0
40 ≤ 45	47.5	4.0	11.9	6	71.3	10	79.8	21.5
45 ≤ 50	64.5	10.5	6.1	4	24.6	12	104.4	33.5
50 ≤ 55	81.0	21.0	3.9	8	30.9	18	135.2	51.5
55 ≤ 60	84.5	22.5	3.8	7	26.3	16	161.5	67.0
60 ≤ 65	133.5	28.0	4.8	10	47.7	24	209.2	91.0
65 ≤ 70	111.5	27.0	4.1	9	37.2	17	246.4	107.5
70 ≤ 75	88.0	28.0	3.1	12	37.7	19	284.1	126.5
75 ≤ 80	87.5	33.5	2.6	11	28.7	11	312.8	137.5
80 ≤ 85	65.5	31.0	2.1	14	29.6	15	342.4	152.0
85 and older	44.5	32.0	1.4	10	13.9	3	*356.3*	*154.5*

(a) Epidemiological Cancer Register of Saarland 12/2010, (b) Regional statistic authorities of German federal state Hesse. (c) Age specific I:M factors multiplied by mortality reported from regional statistic authorities, (d) Breast Cancer Query Database of Marburg-Biedenkopf district, (Incidence) Number of persons per age group on average, (Mortality) Number of breast.cancer mortality per age group on average, (I:M) Incidence-Mortality; IM-Ratio(1996–97)= 131.5/361.7*100=36.4.

IM-Ratio(2003–94)=43.4, (−) No data.

## Discussion

A wide and solid base of evidence is needed for rational health policy decisions. New information technologies can help by collecting all available data and making information relevant to health-policy decisions accessible. Technology may help increase the level of transparency in these decisions. The breadth of evidence considered is especially relevant for age-related diseases such as cancer because incidence rates continue to rise in aging societies [29-31]. This growing trend is also apparent in the regional sample of breast-cancer patients from the clinical register (BCQDB) evaluated in this study. However, this rather crude trend is not sufficient to justify calling the BCQDB an epidemiologically relevant database. Therefore, its accuracy and completeness was assessed.

### Data accuracy

Comparison with external data sources of proven data quality revealed that the BCQDB did not deviate substantially from these proven sources in terms of accuracy. In the period from 1996–97, 5 of 56 indicators (8.9%) showed deviations, and in the years from 2003–04, 8 of 53 indicators (15.1%) deviated systematically. The increase in deviations between the two time periods may be due to a selection bias. Therefore, the verification of the register′s completeness was crucial.

### Data completeness

The raw age-specific incidence curves we calculated showed some fluctuations typical for surveys in rural areas with small numbers of patients [28]. This is especially true for the higher age groups, which include fewer patients as in this study. A more detailed inspection of the cumulative-incidence curves (lifetime-prevalence) also showed possible under-representations for patients older than 75 years (for 1996–97) and 65 years (for 2003–04). This could be interpreted as a sign of selection bias. However, some other details also make this interpretation doubtful. First, the cumulative-incidence curve for the 1996–97 cohort had a “kink” at age 55 years and then ran almost straight and parallel to the comparator up to the higher age groups. This same “kink” was also seen in the 2003–04 cohort, although there it occurred at age 65 years instead of 55 years. This phenomenon points to demographic shifts which are particularly associated with age structure. Therefore, it was necessary to standardize the raw incidence rates to control for age effects.

### Unstandardized and standardized measures

The direct age-standardized incidence rates offset the BCQDB′s deviations from the external data sources that were observed in the raw non-standardized indicators. Particularly for the period from 2003–04, the age-standardized incidence rates exhibited a strong convergence to the reference parameters. This result was confirmed by indirect age-standardization methods, which related raw age-specific incidence rates from the BCQDB to the corresponding demographic population parameters for Saarland. Therefore, it seems reasonable to conclude that the regions′ differing age distributions contributed substantially to the deviations observed in the non-standardized rates. For this reason, estimates of incidence-mortality ratios based on raw age-specific incidence rates and official mortality figures cannot serve as reliable indicators for the assessment of the BCQDB register′s completeness.

### Epidemiological relevance

Based on the assessments of all calculated parameters mentioned above, we conclude that the BCQDB register contains sufficiently accurate, complete, and therefore correct data. It seems safe to assume that the BCQDB is of sufficient epidemiological relevance to allow for further comparisons (e.g. adherence to clinical guidelines, analyses of survival, quality of life, and cost-effectiveness). It can be used for further comparisons and decisions.

### Regional risk adjustments

These conclusions do not contradict the fact that the variables of the data-quality indicator *accuracy* became more divergent over time (increasing to 8 divergent indicators out of 58 for the 2003–04 cohort). The systematic deviations of certain surrogate parameters (e.g. fewer nodal negatives, more small tumors under 2cm) indicate that these increasing divergences may have been caused by the health-care services themselves becoming more effective in the intervening time interval. This interpretation is supported by the increasing use of high quality assured early-detection programs reported elsewhere [32]. To actually measure the regional effects caused by these early-detection programs, high utilization rates of these programs among the target populations are crucial. However, since the civil-register-based invitation systems, and thus their resulting utilization rates, vary greatly between the different German federal states [33], it is methodologically impossible to make a fair and valid comparison of the effectiveness of different regions′ health-care systems and corresponding registers. It is, however, possible to compare different regions′ risk profiles, but any further conclusions regarding a region′s effectiveness would be biased [34]. Overcoming this limitation would require additional data – collected at the individual level and of sufficient quality. This would allow valid comparisons, conclusions, and decisions to be made.

### Harmonization of nomenclature and statistical definitions

Most countries, including Germany, have already taken the first step toward comprehensive (cancer) registers using and offering more epidemiological data by widely implementing information technology in the health-care field [35-37]. The next step is to assess and increase the quality of data in a way that is guided by a theoretical framework such as the GAMOQ. This approach would help minimize doubts concerning the accuracy and completeness of databases. The regional differences displayed by the various external data sources used for comparison in this study highlight how much room there is for interpretation. In particular, harmonizing nomenclatures, statistical distinctions, and reporting certain key parameters of registers or study characteristics could help initiate this process [38], because problems stemming from a data set′s definitional subtleties are difficult to detect. To this end, a number of crucial indicators for survival analysis reports (e.g. year of incidence, years of follow-up etc.) have been proposed in addition to the known pitfalls of cross-country survival analyses [34]. Overall, all these efforts attempt to facilitate valid comparisons and rational decisions.

### Methodological strengths

The strength of this study was the approach that allowed us to integrate the many different available data sources and use them to estimate the epidemiological relevance of the BCQDB. Doing so is especially advantageous for small-scale population-based databases or for clinical registers of rural areas. The reason we chose breast cancer as the exemplary disease with which to demonstrate this procedure was because there are many publicly available sources of breast-cancer data. However, the procedure used in this study which is only one part of the GAMOQs is also applicable to any other disease. It goes beyond the usual comparison of epidemiological measures and gives a deeper understanding of the relationship between a data set′s accuracy and completeness. At this point it is worth emphasizing the analytic value of non-standardized measures, which in this case allowed regional differences and other irregularities to be identified [22] before beginning the statistical analysis. Such qualitative insights are of particular interest before data has been analyzed and interpreted as they can inform the analysis. In the case of the BCQDB, the assessments of accuracy and completeness show that future analyses should carefully conclude and interpret data results particularly if patients are older than 65 or 75 years (depending on the cohort in question).

### Methodological shortcomings

A general weakness of clinical registers working with complete surveys that are administered only to utilizers of regional breast cancer networks is the fact that selection bias can occur [9]. Therefore, completeness-estimation techniques such as the incidence-mortality ratios (IMR) were developed to quantify to what extent the clinical register did not capture missing cases. The IMR approach belongs to the historical methods and a common threshold of 90% or more has been established to consider a register as complete [16,26]. However, in the context of a small-area analysis using time-interval-bounded incidence data as in this study, it is not possible to use incidence mortality ratios (IMR) in the usual way to estimate the size of target population not included in the data. This is because the already ill prevalent patients who died in the observed time intervals (1996–97, 2003–04) were excluded from the BCQDB-register by definition, and were thus not captured in the mortality rates. These cases *are* however counted in the official mortality statistics taken from the official statistical authorities, and thus figure in to the “expected cases” factor in the denominator of the IMRs. This leads to overestimated IMRs and thus to a higher percentage of patients dying in a given time interval. Therefore, our data-incompleteness estimates – which estimated the number of cases missing from the data at 64% and 57% – were unrealistically high. Finally, the IMR approach also entails all of the usual drawbacks associated with disease-specific cause-of-death statistics and postmortem examinations [39,40].

### Future potential of benchmarks

Further methodological developments for estimating the correctness (e.g. accuracy, completeness) of data sets and cohort studies will be necessary in the future, both in order to allow researchers to gain access to the information that remains hidden in the growing amount of data and to allow this data to be exploited for epidemiologically and health-economically valid comparisons, conclusions, and decisions. There is demand for such new methodologies due to infrastructural changes (such as integrated health-care networks, organ centers, and clinic chains) which are shifting the focus to multidisciplinary oriented health care for chronic diseases. Furthermore, these new organizational developments are population-based and cover countries, states, districts, and counties. This means that demographic distributions are available from statistical authorities, which facilitates the estimation of incidence rates. Therefore, the completeness estimation for a clinical register in question is restricted to patients with permanent residence of the corresponding area and is not valid for non-resident patients with the same disease. Under these circumstances, health-care providers can be compared to external benchmarks. This approach is superior to anonymous benchmarks based on averages, because it allows much more than the usual comparison of a few abstract quality indicators [41,42]. Specifically, it allows the best performers on a given quality indicator to be used as the benchmark, enabling conclusions to be drawn regarding how the benchmark organization achieved its results as well as how similar results could be achieved at the lower-performing organization. However to attain such external quality assurance mechanisms, it is essential to identify comparable organizations or institutions which can be used as peer groups.

## Conclusion

In this study, we proposed accuracy statistics that rely on patients′ basic characteristics and common risk, prognosis, and predictive factors. However, the list of indicators could be more extensive to 1) describe the general conditions of health care institutions and their patients which were exposed to new performance-enhancing quality tools (such as quality-management systems) and 2) to compare and decide whether new structural or process-related changes lead to expected improvements. This approach helps to reproduce the results gained in some (specialized) health care institutions. It fosters the understanding why some innovations have worked and others have not and how they are applicable to non-specialized institutions [43-45]. Therefore, only the comparison between “equal” institutions will allow for valid comparisons, conclusions, and sound decisions that will lead to further improvements in the quality and transparency of public health care.

## Competing interest

The authors declare that they have no competing interests.

## Authors’ contribution

COJ contributed substantially to the conception, acquisition of data, design, analysis and interpretation of data, drafting the manuscript, and gave final approval. MK was involved in the acquisition of data, contributed substantially to the discussion, revised and approved the final draft. UW was involved in the acquisition of data, contributed substantially to the discussion, revised and approved the final draft. USA was involved in the conception, analysis, and interpretation of data, revised critically for important intellectual content, and gave approval for the final draft. All authors read and approved the final manuscript.
